# A Comprehensive Assessment of Food Parenting Practices: Psychometric Properties of the Portuguese Version of the HomeSTEAD Family Food Practices Survey and Associations with Children’s Weight and Food Intake

**DOI:** 10.3390/ejihpe10010032

**Published:** 2020-02-05

**Authors:** Lisa Afonso, Joana Castro, Nuno Parente, Sandra Torres

**Affiliations:** 1 Center for Psychology, Faculty of Psychology and Educational Sciences, University of Porto, Rua Alfredo Allen, 4200-135 Porto, Portugal; storres@fpce.up.pt; 2 Viver Mais Family Health Unit, Maia-Valongo Health Centre Group, Avenida Luís de Camões, n.º 290, 3.º andar, 4474-004 Maia, Portugal; joanacastro@hotmail.it (J.C.); nunomiguelpm@gmail.com (N.P.)

**Keywords:** feeding practices, children, factor analysis, weight, dietary intake, parenting

## Abstract

The Home Self-Administered Tool for Environmental Assessment of Activity and Diet (HomeSTEAD) survey evaluates a broad spectrum of food parenting practices related to parental use of control, autonomy support, and structure. This study aims to test the psychometric properties of the Portuguese version of the HomeSTEAD family food practices survey in parents of 3–12 year old children. Data were collected from 184 parents/caregivers. We performed an exploratory factor analysis (EFA), calculated the internal consistency coefficients of each subscale, and tested for associations with children’s food intake and weight. Based on the EFA, 61 items were included in the Portuguese version of the HomeSTEAD family food practices survey, and were distributed among four Coercive Control Practices (16 items); five Autonomy Support Practices (17 items); and nine Structure Practices (28 items). All scales demonstrated an acceptable level of internal consistency. A higher body mass index (BMI) SD score in children was associated with higher levels of restriction and weight talk by parents and distractions during meals. Higher levels of distractions during meals were also associated with higher sweets intake in children. Additionally, higher levels of parental modeling and the establishment of rules and limits were associated with lower intake of sugar-sweetened beverages. These associations provide preliminary evidence of the HomeSTEAD family food practices survey’s construct validity and attest to its potential to assess parental strategies and provide useful information to improve children’s eating.

## 1. Introduction

Parents are the main mediators between children and food. They guide their dietary intake and eating behavior through strategies called food parenting practices. The study of food parenting practices has traditionally focused on practices of control, namely restriction and pressure to eat [1]. However, as interest in the field grew due to its potential to prevent obesity, instruments were created to study alternative approaches to control [1]. For instance, those food parenting practices that are concerned with the organization of the food environment in terms of availability of healthy foods, and positive strategies that parents can apply to promote healthy eating, such as nutrition education, have more recently been taken into consideration [2]. However, inconsistencies remained in the terminology and definitions and a comprehensive measure was lacking [3].

Recently, Vaughn and colleagues mapped and organized the food parenting practices documented in the literature [4]. The Home Self-Administered Tool for Environmental Assessment of Activity and Diet (HomeSTEAD) survey [5] is composed of different measures of the home environment, including one instrument for the assessment of the social food environment that resulted from a content map of food parenting practices. The HomeSTEAD family food practices survey captures 24 food parenting practices that are organized into three higher-order food parenting groups, namely structure, control, and autonomy support. This survey is aligned with Self-Determination Theory (SDT), and applies concepts from SDT to research on parenting [6].

According to SDT, providing structure to children implies that parents clearly communicate rules and expectations to children, give children confidence, and provide competence-relevant feedback during the process [6]. Structure is seen as ‘the what’ of food parenting practices. This consists not only of the organization of the food environment but also the establishment, communication, and monitoring of clear and consistent rules and guidelines about food intake, meal settings, and family eating habits. Some of these practices have been associated with better eating habits in children [7,8,9]. Establishing rules and limits on unhealthy foods and drinks seems to effectively prevent their intake [10]. Greater availability and accessibility of fruit and vegetables lead to higher intake of these foods in children [10]. On the other hand, a more permissive parenting style, with less guided choices and that allows a child to decide when, what, and how much to eat, was associated with a higher weight status in children [3]. Furthermore, this was associated with lower intake of fruit and vegetables [11] and, in general, lower dietary quality in low-income households [12]. Distractions while eating were shown to be associated with a poorer-quality diet in children, particularly a higher intake of high-fat and high-sugar foods [13,14], and, in general, a larger amount of ingested food [15]. It is also important to note that the concept of ‘structure’ is related to the coherence of established rules and, thus, parental modeling is an important indicator of it [6]. A parental modeling effect was associated with a better-quality diet in children [8].

If structuring a food environment is ‘the what’ of food parenting practices, the strategies used by parents to persuade children to eat the available food can be seen as ‘the how’. According to SDT, to motivate children to act, parents can use practices of discipline, which lead to controlled motivation, or practices that support autonomy, which impel autonomous motivation [4,6]. Coercive control of food parenting practices has a parent-centered nature and some counterproductive effects of its use are documented in the literature [1]. For example, parental attempts to exert control over the quality of food, the quantity of food, and the time when food is eaten undermine the capacity of children to self-regulate their eating. Previous longitudinal studies support this finding, and show a positive bidirectional association between parental restrictions on eating and higher appetite disinhibition behaviors and weight status in children [16,17,18]. Thus, restriction seems to be a counterproductive response to children who are overweight. Studies also indicated that parents tend to exert pressure to eat in response to their child having a lower weight status and that this strategy is also counterproductive, leading to a longitudinal decrease in the body mass index (BMI) [16,17,18].

Regarding practices that support autonomy, according to SDT, autonomously regulated behaviors are intrinsically motivated in or perceived as personally meaningful by children [19]. Autonomy support consists in giving choices to children, taking into account their perspective, and providing a clear rationale when a choice is constrained [6]. This can foster self-governed motivation and competence in children [6]. Looking specifically to food parenting practices, practices that support uncontrolled behavior may be characterized by caregiver guidance and training while respecting children’s preferences and their hunger and satiety cues [20]. Feeding practices that support autonomy, such as nutritional education and encouragement, have been shown to improve the selection and intake of fruit and vegetables [8]. This is a relatively new concept in food parenting practices research, and more studies are needed to understand how these practices can be alternatives to control and positively influence children’s food intake and eating behavior [21].

The HomeSTEAD family food practices survey is a promising measure for the comprehensive evaluation of food parenting practices. It includes a broad spectrum of parenting practices in addition to the focus on ‘Restriction’, ‘Pressure to eat’, and ‘Monitoring’ as addressed in the Child Feeding Questionnaire [22]. Another well-known comprehensive measure of food parenting practices, the Comprehensive Feeding Practices Questionnaire [23], can already be used to evaluate how parents model, teach, or encourage healthy eating. However, the HomeSTEAD family food practices survey integrates a broader evaluation of the concepts of ‘Structure’ (with subscales such as distractions and rules and limits) and ‘Support of Autonomy’ (with subscales such as guided choices). However, attempts to develop this more comprehensive measure require further research on construct validity and reliability in varying linguistic and cultural contexts [21]. Therefore, we sought to investigate the psychometric properties of a Portuguese translation of the HomeSTEAD family food practices survey in parents of 3–12 year old children. We tested the factorial structure of each group of practices independently, following the same procedure that was adopted in the study with the original version. Additionally, we tested the construct validity by analyzing the association with children’s weight (BMI SD score and weight status) and food intake. Based on the described findings, we expected a positive correlation between children’s weight and the ‘Restriction’ and ‘Weight talk’ subscales, and a negative correlation with ‘Pressure to eat’ [16,17,18]. Regarding children’s intake, a poor-quality diet (high-fat and high-sugar food and drink intake) was expected to be negatively associated with the ‘Rules and limits’ [10] and ‘Guided choices’ [3] subscales and positively associated with the ‘Distractions’ subscale [13,14]. On the other hand, a better-quality diet (higher fruit and vegetables intake) was predicted to be negatively associated with the ‘Guided choices’ subscale [11] and positively associated with the ‘Availability and accessibility of fruit and vegetables’ [10], ‘Nutritional education’, ‘Encouragement’, and ‘Modeling’ subscales [8]. Given the lack of empirical data regarding the other subscales of the HomeSTEAD family food practices survey, no significant correlations were expected, with the exception of two subscales. Based on theoretical reasoning, we expected to find an association between the ‘Planning and preparation of healthy meals’ and ‘Praise’ subscales and higher fruits and vegetables intake [24].

## 2. Materials and Methods

### 2.1. Participants

The participants included 184 parents or caregivers of children aged 3–12 years. Participants were recruited from December 2017 to September 2018 in two different contexts. One context was a health care center, where parents were invited sequentially to participate by family doctors and nurses during child health care visits (*n* = 76). The other context was 11 parents’ associations of preschools and primary schools that invited parents to answer the questionnaire (*n* = 108). Parents or caregivers were eligible to participate if they were ≥18 years old and if they were involved in the management of a child’s eating habits (an involvement score of ≥5 on a 0–10 rating scale, from ‘Not at all’ (0) to ‘Extremely involved’ (10), regarding food acquisition and meal planning and preparation). Furthermore, parents or caregivers were not eligible to participate if the child whose eating habits were under management had any disability that required regular medical care or specific food habits or had had nutritional counseling in the previous 6 months.

### 2.2. Procedures

This study complied with the Ethical Principles of the Declaration of Helsinki [25] and was approved by the Ethics Committee of the Faculty of Psychology and Educational Sciences of the University of Porto (reference number 2017/10-4).

To adapt the HomeSTEAD family food practices survey to Portuguese, we followed the recommended guidelines for cross-cultural adaptation of instruments [26]. Permission for translation, adaptation, and validation was obtained from the authors. In the translation process, some cultural adjustments were needed, mainly in relation to foods and cooking methods (e.g., in the item ‘When you serve potatoes, how often are they fried, like French fries or hash browns?’, we dropped the examples, because they are not common in Portuguese culture). We applied the back-translation technique to find a preliminary version and tested it with five volunteers. These volunteers satisfied the eligibility criteria of our sample and were from different sociodemographic backgrounds. We asked each of them what the meaning of each item was and made adjustments when needed to improve comprehension.

Questionnaires were self-administered in an electronic format. The exception was the Food Frequency Questionnaire, which was only answered at the health care center with the support of a professional dietician. Therefore, data on food intake were not collected from parents that were not evaluated face-to-face (*n* = 75). Additionally, we assumed that data were missing in anthropometrics terms if the data relative to the last measurement in a child were missing or if they had been collected more than three months ago (*n* = 40).

### 2.3. Measures

#### 2.3.1. The HomeSTEAD Family Food Practices Survey

The HomeSTEAD family food practices survey is an 86-item questionnaire that evaluates 24 food parenting practices and is organized into three main domains: coercive control, structure, and autonomy support. Coercive practices, which reflect parents’ attempts to impose their will, are measured with 16 items that are grouped into five subscales: restriction, threats and bribes, soothing with food, a “clean plate” policy, and pressure to eat. Structure practices, which reflect parents’ organization of a child’s environment, are measured with 46 items that are grouped into 12 subscales: monitoring of unhealthy foods, rules and limits around unhealthy foods, components of the child’s eating environment (i.e., meal setting, family eating, atmosphere of meals, eating area, and distractions), planning and preparation of healthy meals, attractive presentation of healthy foods, availability and accessibility of healthy foods, modeling, and weight talk. Autonomy support practices, which reflect the support of child competence and self-endorsement of behaviors, are measured with 24 items that are grouped into seven subscales: encouragement, reasoning, praise, nutrition education, and guided choices around when, what, and how much food is eaten. Parents answered each item using a five-point Likert scale (with the exception of one item that was answered in a seven-point scale) [5].

#### 2.3.2. Food Intake

Parents/caregivers answered a Food Frequency Questionnaire [27], which rated the frequency of their children’s intake of a list of foods and drinks within a nine-point scale that ranged from “never” to “three or more times a day”. Portions of a food group’s intake (Fruit and Vegetables, Sugar-Sweetened Beverages, and Snacks and Sweets) resulted from the sum of frequencies of the items that composed each group.

#### 2.3.3. Child’s Anthropometric Data

Parents/caregivers reported data contained in their child’s health record booklet related to the last anthropometric measurement (date, weight, and height). For those recruited in child health care visits (*n* = 76), the child’s anthropometrics were evaluated just before the interview. We computed the BMI as the value of weight (kg) over the squared height (m), and the age- and sex-specific BMI SD score (BMIz) according to World Health Organization (WHO) Growth References [28]. We defined a child’s weight status (underweight, normal weight, overweight, and obesity) based on the cut-offs of the WHO [28].

### 2.4. Statistical Anaysis

The assumption of normality was confirmed by a Kolmogorov–Smirnov test and by analyses of each item distribution by the skewness and kurtosis values [29]. We excluded items with high values of skewness and kurtosis (|Sk| > 3 and |K| > 7). We then performed an exploratory factor analysis (EFA) to test the dimensionality of items. We ran separate EFAs for each group of practices (control, structure, and autonomy support), similarly to the procedure that was adopted in the original study, assuming the distinctiveness of the constructs [5]. We confirmed the factorability of data with Bartlett’s test of sphericity (*p* < 0.05) and the Kaiser–Meyer–Olkin (KMO) measure of sampling adequacy (≥0.6). To obtain a factor’s loading structure, we applied an orthogonal rotation (VARIMAX). To determine the number of dimensions, the criterion of an eigenvalue greater than 1, the scree test, and a parallel analysis were used [30]. We identified and excluded items with low factor loadings (<0.40) and then repeated the analysis.

Internal consistency coefficients (Cronbach’s Alpha) for each subscale were held to reflect an acceptable level of internal consistency at values over 0.60, considering the reduced number of items in each factor [29]. Inter-item correlations were expected to be at values over 0.30, and we calculated the mean inter-item correlation (MIIC), which was expected to be between 0.15 and 0.50 [31]. For subscales with an MIIC higher than 0.50, multicollinearity between items was tested by variance inflation factors (<4.0) and tolerance (>0.2) [29].

The final score of each subscale was calculated by the mean of the items that composed it, with higher punctuations reflecting higher levels of each practice. The exception was ‘Guided choices’, in which higher levels reflected a lower level of guidance by parents and a higher degree of autonomy for the child concerning when, what, and how much to eat. Therefore, the associations to be expected with this subscale should be read inversely.

We tested the construct validity by analyzing the Pearson’s correlations of the score of each parental practice with children’s BMIz and food intake (Fruit and Vegetables, Sugar-Sweetened Beverages, and Snacks and Sweets). Additionally, we tested differences in scores between weight status and specific groups with one-way ANOVA.

All analyses were performed with the IBM^®^ SPSS^®^ Software, version 24.0 [32].

## 3. Results

### 3.1. Participants

Participants were mothers (*n* = 170; 92.4%), fathers (*n* = 11; 6.0%), grandmothers (*n* = 2; 1.1%), and a stepmother (*n* = 1; 0.5%). Characteristics of participants and their children are presented in Table 1.

### 3.2. Factorial Structure of the Portuguese Version of the HomeSTEAD Family Food Practices Survey

#### 3.2.1. Coercive Control Scales

All items had satisfactory factor loadings. The EFA identified four factors, which were confirmed by the parallel analysis to explain 61.3% of the variance. Three factors (‘Restriction’, ‘Soothing with food’, and ‘“Clean plate” policy’) remained the same as in the original scale (see Table 2). Items from the other two original subscales, ‘Pressure to eat’ and ‘Threats and bribes’, were punctuated together in the same factor that we renamed to ‘Threats, bribes, and pressure to eat’. All subscales of Coercive Control had a good level of internal consistency, with acceptable coefficients (between 0.67 and 0.81) and MIIC (between 0.39 and 0.50).

#### 3.2.2. Autonomy Support Scales

We excluded seven items, and the reasons for exclusion per item are provided in Appendix A. Afterwards, we repeated an EFA with the remaining 17 items. All items had sufficient factor loadings. We found five factors, explaining 61.7% of the total variance. Two factors (‘Praise’ and ‘Guided choices: amount of food eaten’) replicated the original subscales of the HomeSTEAD family food practices survey (see Table 3). The factor ‘Nutrition education’ was very similar to that in the original subscale, with only one excluded item. We excluded one item of the original subscale ‘Guided choices: when food is eaten’ and another of the original subscale ‘Guided choices: what food is eaten’. The remaining items of those subscales were punctuated together in the same factor, which was therefore called ‘Guided choices: when and what food is eaten’. We excluded three items of ‘Reasoning’ and one of ‘Encouragement’. The remaining items of those subscales were punctuated together in the same factor that we renamed ‘Encouragement and reasoning’.

All final subscales had an acceptable level of internal consistency (between 0.62 and 0.73) and an acceptable MIIC (between 0.31 and 0.50).

#### 3.2.3. Structure Scales

We excluded 18 items; the reasons for exclusion per item are provided in Appendix A. We repeated the EFA with the remaining 28 items, which revealed the presence of nine factors, explaining 66.9% of the total variance. The scree plot and the parallel analysis confirmed this nine-factor structure. Three (‘Monitoring of unhealthy foods’, ‘Weight talk’, and ‘Distractions’) of the nine factors replicated the original scale (see Table 4). The factors ‘Atmosphere of meals’ and ‘Availability and accessibility of healthy foods’ were very similar to the original subscales (with only one excluded item per subscale). Five of the excluded items represented the factors ‘Meal setting’ and ‘Family eating’ that, therefore, did not emerge. Only one factor of ‘Meal setting’ persisted and was punctuated with ‘Rules and limits’. As this was theoretically related to the factor ‘Rules and limits’, it was included in this subscale, which in turn remained similar to the original one (with only one excluded item). Compared to the original subscales, the factors ‘Eating area/physical space’ and ‘Modeling’ had two and three excluded items, respectively. Major differences were found in the subscale ‘Planning and preparation of healthy meals’, with four deleted items. The other two items were punctuated together with the remaining item of ‘Attractive presentation of healthy foods’ (the other two were excluded) and the remaining item of ‘Availability and accessibility of healthy foods’. We considered these items to be theoretically related and we renamed this factor ‘Planning, preparation, and attractive presentation of foods and meals’.

The structure subscales of the Portuguese version of the HomeSTEAD survey had an acceptable level of internal consistency (between 0.61 and 0.94) and MIIC (between 0.26 and 0.85). We confirmed that there was no multicollinearity between items of subscales with an MIIC of over 0.50.

In total, the Portuguese version of the HomeSTEAD family food practices survey ended up with 61 items: 16 items representing four Coercive Control Practices, 17 items representing five Autonomy Support Practices, and 28 items representing nine Structure Practices.

### 3.3. Construct Validity

The differences between food parenting practices in terms of children’s weight status categories are reported in Table 5. We also present correlations between the score of each food parenting practice of the HomeSTEAD family food practices survey and children’s BMIz.

Children’s BMIz was strongly correlated with ‘Restriction’ (*r* = 0.48; *p* < 0.001). When evaluating the differences between weight statuses, parents reported higher levels of restriction when their children had overweight (*M* = 2.6; SD = 1.0) and obesity (*M* = 3.1; SD = 1.1) compared to parents of children of normal weight (*M* = 1.8; SD = 0.8). Children’s BMIz was moderately correlated with ‘Weight talk’ (*r* = 0.36; *p* < 0.001), with higher levels of this practice reported by parents of children with obesity (*M* = 3.1; SD = 1.2) compared to parents of children with overweight (*M* = 2.5; SD = 0.9) and normal weight (*M* = 2.2; SD = 0.9). Additionally, children’s BMIz was moderately correlated with ‘Distractions’ (*r* = 0.31; *p* < 0.001), with higher levels of this practice reported by parents of children with obesity (*M* = 3.9; SD = 1.1) compared to parents of children with overweight (*M* = 3.3; SD = 1.3) and normal weight (*M* = 2.9; SD = 1.1).

Weak negative correlations were found between ‘Guided choices: when and what food is eaten’ and BMIz (*r* = –0.17; *p* < 0.05).

Correlations between the score of each food parenting practice of the HomeSTEAD family food practices survey and food intake are reported in Table 6, as a means to analyze construct validity.

Children’s sweets intake was positively and moderately correlated with ‘Distractions’ (*r* = 0.25; *p* < 0.001). Children’s sugar-sweetened beverages intake was negatively and strongly correlated with ‘Modeling’ (*r* = –0.40; *p* < 0.001) and ‘Rules and limits’(*r* = –0.45; *p* < 0.001) and moderately correlated with ‘Guided choices: when and what food is eaten’ (*r* = 0.21; *p* < 0.001). Weak correlations were found between children’s snacks intake and ‘Soothing with food’ (*r* = 0.19; *p* < 0.05), ‘Weight talk’ (*r* = 0.19; *p* < 0.05), ‘Modeling’ (*r* = –0.20; *p* < 0.05), and ‘Rules and limits’ (*r* = –0.20; *p* < 0.05).

## 4. Discussion

This study aimed to investigate the factor structure and psychometric properties of a Portuguese version of the HomeSTEAD family food practices survey in a sample of parents of children aged 3–12 years old.

The Portuguese version of the HomeSTEAD family food practices survey is composed of 18 scales with an acceptable level of internal consistency. A total of 25 items were removed from the Portuguese version and, consequently, two factors were eliminated: ‘Family eating’ and ‘Meal setting’. The majority of the excluded items were highly dependent on culture. For instance, to the question ‘what do family dinners on weekdays look like at your home’, almost all parents answered ‘family members sit down and eat together’. The scale with the most excluded items was ‘Planning and preparation of healthy meals’, and we should acknowledge that it was also the scale we made major cultural adjustments to during the adaptation to Portuguese, mainly in relation to foods and cooking methods.

Regarding construct validity and unlike the original study, we found significant correlations between some food parenting practices and children’s BMIz. Parents of children with a higher BMIz reported higher levels of restriction. Comparing the scores of the practices by children’s weight statuses, parents of children with overweight and obesity revealed higher scores than parents of children with a normal weight for their age. This finding is in line with those of previous studies on restriction [16,17,18]. We should also note that the ‘Restriction’ questions of the HomeSTEAD family food practices survey are relevant to weight control (e.g., “How often do you restrict your child’s food intake so that she or he will not gain weight?”) compared to the ‘Restriction’ questions from other existing instruments, such as the Child Feeding Questionnaire (e.g., “If I did not guide or regulate my child’s eating, she would eat too much of her favorite foods”). Therefore, this positive association between restriction and weight is even more likely using the HomeSTEAD family food practices survey and this measure may be more useful for evaluating restrictions related to weight control. We also found that ‘Weight talk’ was higher in parents of children with obesity compared with parents of children with overweight or children with a normal weight for their age. This finding was also in the predicted direction [16,17,18]. We did not find a significant correlation between BMIz and the parent’s use of pressure to eat, which might be explained by the fact that this subscale in the Portuguese version also integrates items related to threats and bribes.

Regarding the practices of coercive control, the only correlation found with children’s intake was between the use of soothing with food and children’s snacks intake. Previous studies have already observed an association between emotional feeding and children’s snacking [33]. To date, this specific practice of ‘Soothing with food’ has mostly been studied in infants, and a longitudinal association with a higher weight has been reported [34]. This scale of the HomeSTEAD family food practices survey adds the possibility to evaluate this specific practice in parents of older children.

Concerning the construct validity of autonomy support practices, the only practice that correlated with children’s BMIz and food intake was guided choices. Lower levels of parental guidance in relation to what and when to eat were associated with a lower BMIz in children. This was not in the direction initially predicted, as some studies have found that a more permissive parenting style (with less guidance by parents on children’s choices) is associated with a higher weight status [3]. However, this negative association may be related to a reverse causality, i.e., parents may be giving a higher degree of autonomy to children in relation to food intake as they have a lower BMIz. In fact, some studies have suggested that parents tend to apply strategies to monitor and control eating in response to children’s weight [35,36,37], and our findings point in the same direction with less guidance associated with a lower BMIz. Additionally, concern about a child with overweight was found to be positively associated with healthy eating guidance, as evaluated by the Comprehensive Feeding Practices Questionnaire in a previous study [38]. On the other hand, a lower number of parent-guided choices was associated with sugar-sweetened beverages intake in our sample [3]. However, we did not find any association with snacks, sweets, or fruit and vegetables intake.

Regarding the structure practices, we found positive correlations between being distracted during meals and children’s BMIz. As expected [13,14], those distractions were also correlated with a worse-quality diet by a higher snacks and sweets intake. However, we did not find any correlations with sugar-sweetened beverages intake, in line with previous studies [13,14,15]. Recent studies have shown positive associations between daily television watching and children’s weight [39], and between children watching advertising during a meal and higher intake of poor-quality food [40]. What this scale adds is the possibility to evaluate the specific practice of parents leaving the television turned on during meals, thus allowing for this practice to be assessed. Concerning correlations between structure practices with children’s diet, we found that a higher degree of establishment of rules and limits by parents was associated with a lower intake of snacks, sweets, and sugar-sweetened beverages. These were the expected directions of associations [10]. Additionally, a higher degree of reported modeling by parents was associated with lower sugar-sweetened beverages intake, which is also supported by the literature [8].

We did not observe the expected correlations between ‘Availability and accessibility of healthy foods’, ‘Nutrition education’ ‘Encouragement’, ‘Planning and preparation of healthy meals’, and ‘Praise’ with fruit and vegetables intake. Some associations between practices of parents and children’s weight and food intake may not have been found because the questionnaire targeted a large age range. The age ranges of 3–6 and 6–12 years are characterized by different levels of autonomy and parental and peer influence, which affect children’s behaviors differently [10,41].

The strength of this study is our attempt to make the HomeSTEAD family food practices survey available for use in research on food parenting practices, considering that it covers a broad spectrum of practices. Additionally, it adds knowledge on the psychometric properties of the HomeSTEAD family food practices survey. This study has also some limitations that deserve attention. First, we used the minimum acceptable sample size to analyze the factor structure. Second, the food intake assessment was based on a self-report by parents, without an observational assessment of the home’s food environment (as performed in the original study) or of meals. Despite this, the Food Frequency Questionnaire has been shown to be a good instrument to evaluate dietary intake in children, with strong correlations with data from food diaries and serum biomarkers [27]. Regarding anthropometrics, we asked parents to report data collected by clinicians as an assurance of quality. However, it would have been useful to measure objectively the height and weight of each child in order to avoid excluding those with less-current measurements or parents without access to their child’s health record booklet. Third, the majority of the participating parents were mothers; despite this, we attempted to obtain answers from both mothers and fathers. Whenever we had both parents present, the mother was mostly asked to answer because of her greater involvement in the management of infant feeding. This problem is common to other studies on food parenting practices [42], and limited our ability to evaluate precisely the food parenting practices that were exerted on the child, as meal time observations have indicated that fathers’ practices differ from mothers’ [43].

In the future, the factor structure of the Portuguese version of the HomeSTEAD family food practices survey should be tested in a larger sample. There is also a need to test for invariance in children’s age and gender [42]. It would also be important to confirm the temporal stability with a test–retest reliability analysis.

## 5. Conclusions

In conclusion, the HomeSTEAD family food practices survey, which evaluates food parenting practices of Coercive Control, Structure, and Autonomy Support, was adapted to be used with Portuguese parents of children aged 3–12 years old. This study supports the view that some items need to be reformulated during the adaptation of this survey to countries with different cultural habits, namely those related to foods and cooking methods and meal settings. We found significant correlations between food parenting practices, identified in the literature as closely linked to obesity and children’s BMIz and food intake, which provide preliminary evidence of the construct validity of the Portuguese version of the HomeSTEAD family food practices survey.

## Figures and Tables

**Table 1:** Characteristics of parents/caregivers and children (n = 184).

Parents/Caregivers		
Sex	Female, n (%)	173 (94.0)
	Male, n (%)	11 (6.0)
Age (years), mean (SD)		37.0 (6.1)
With university degree, n (%)		92 (50.0)
**Children**		
Sex	Female, n (%)	88 (47.8)
	Male, n (%)	96 (52.2)
Age (years), mean (SD)		6.4 (2.5)
Weight status, n(%) ^1^	Underweight	0 (0.0)
	Normal weight	92 (63.9)
	Overweight	30 (20.8)
	Obesity	22 (15.3)

^1^ Missing data for 40 participants.

**Table 2:** The factor loadings and internal consistency of Coercive Control Practices of the Home Self-Administered Tool for Environmental Assessment of Activity and Diet (HomeSTEAD) family food practices survey for parents of 3–12 year old children *(n* = 184).

Portuguese Version of Coercive Control Scales		Original Factor	Factor Loadings
**Restriction** (α = 0.78; MIIC = 0.50)	How often do you restrict (or try to restrict) your child’s food intake so that she or he will not gain weight? ^1^	Restriction	0.631
	How often do you tell your child not to eat something because it will make him or her fat? ^1^	Restriction	0.914
	How many times have you told your child to eat less food or eat different foods to lose weight or to keep from gaining weight? ^1^	Restriction	0.911
**Soothing with food** (α = 0.77; MIIC = 0.46)	I give my child something to eat or drink when she or he is bored or worried, even if I know she or he is not hungry. ^2^	Soothing with food	0.773
	Offering my child something to eat is one of the best ways to stop his or her temper tantrums. ^2^	Soothing with food	0.899
	How often do you use food as a way to distract your child (e.g., if he or she is preventing you from doing your chores)? ^1^	Soothing with food	0.515
	To get my child to behave himself or herself I promise him or her something to eat. ^2^	Soothing with food	0.845
**“Clean Plate” Policy** (α = 0.67; MIIC = 0.39)	This is a police in our home…		
	If my child puts food on his or her plate, he or she has to eat it. ^2^	“Clean plate” policy	0.887
	My child must stay at the table until a specified amount of food has been eaten. ^2^	“Clean plate” policy	0.619
	My child is expected to eat everything on his or her plate at dinner. ^2^	“Clean plate” policy	0.876
**Threats, Bribes, and Pressure to eat** (α = 0.81; MIIC = 0.41)	If your child does not like something, how often do you tell him or her that he or she will get a dessert if he or she tries it? ^1^	Threats and Bribes	0.621
	How often do you promise your child something other than food (e.g., toy or favorite activity) in return for eating specific foods? ^1^	Threats and Bribes	0.539
	I have to punish or remove privileges to get my child to eat more. ^2^	Threats and Bribes	0.784
	How often do you help feed your child (e.g., hold the spoon to put food into his or her mouth)? ^1^	Pressure to eat	0.761
	How often do you beg your child to eat? ^1^	Pressure to eat	0.795
	How often do you coax or sweet talk your child to get him or her to take a bite? ^1^	Pressure to eat	0.832

^1^ Response options offer a five-point scale: never, rarely, sometimes, often, and always. ^2^ Response options offer a five-point scale: strongly disagree, disagree, neutral, agree, and strongly agree. α = Cronbach’s α; MIIC = mean inter-item correlation; and (R) = items that require reverse coding.

**Table 3:** Factor loadings and Internal Consistency of Autonomy Support Practices of the HomeSTEAD family food practices survey for parents of 3–12 year-old children (N = 184).

Portuguese Version of Autonomy Support Scales		Original Factor	Factor Loadings
**Encouragement and reasoning** (α = 0.73; MIIC = 0.50)	I encourage my child to look forward to the meal. ^1^	Encouragement	0.830
	I encourage my child to enjoy his or her food. ^1^	Encouragement	0.860
	I reason with my child to get him or her to eat. ^1^	Reasoning	0.569
**Praise** (α = 0.67; MIIC = 0.42)	I praise my child if he or she eats what I give him or her. ^1^	Praise	0.712
	I praise my child if she or he eats a new food. ^1^	Praise	0.850
	I praise my child for choosing a healthy snack. ^1^	Praise	0.689
**Nutrition Education** (α = 0.68; MIIC = 0.41)	How often do you try to make foods more familiar to your child by telling him or her where it came from? ^2^	Nutrition Education	0.514
	I discuss with my child the nutritional value of foods. ^1^	Nutrition Education	0.856
	Do you give your child reasons for the rules you make about food and eating? ^2^	Nutrition Education	0.816
**Guided Choices: When and what food is eaten** (α = 0.62; MIIC= 0.31)	I let my child eat between meals whenever she or he wants. ^1^	Guided Choices: When Food Is Eaten	0.697
	I let my child decide when he or she would like to have his or her meal. ^1^	Guided Choices: When Food Is Eaten	0.650
	I allow my child to choose what she or he has for snacks. ^1^	Guided Choices: What Food Is Eaten	0.544
	I decide what my child eats between meals. ^1^ (R)	Guided Choices: What Food Is Eaten	−0.691
**Guided Choices: Amount of food eaten** (α = 0.65; MIIC = 0.32)	During meals, I allow my child to decide when she or he has had enough to eat. ^1^	Guided Choices: Amount of food eaten	0.628
	At snack time, I allow my child to decide when she or he has had enough to eat. ^1^	Guided Choices: Amount of food eaten	0.466
	I know better than my child does if she or he is hungry or full. ^1^ (R)	Guided Choices: Amount of food eaten	−0.751
	When your child says “I’m not hungry,” how often do you reply “You need to eat anyway”? ^2^ (R)	Guided Choices: Amount of food eaten	−0.679

^1^ Response options offer a 5-point scale: strongly disagree, disagree, neutral, agree, and strongly agree. ^2^ Response options offer a 5-point scale: never, rarely, sometimes, often, and always. α = Cronbach’s α; MIIC = Mean inter-item correlation; and (R) = items that require reverse coding.

**Table 4:** Factor loadings and Internal Consistency of Structure Practices of the HomeSTEAD family food practices survey for parents of 3–12 year-old children (N = 184).

Portuguese Version of Structure Scales		Original Factor	Factor Loadings
**Monitoring of Unhealthy Foods** (α = 0.94; MIIC = 0.85)	I keep track of (either in my head or written down)...		
	The sweets (candy, ice cream, cake, pies, pastries) that my child eats. ^1^	Monitoring	0.927
	The snack food (potato chips, cheese puffs) that my child eats. ^1^	Monitoring	0.966
	The sugary drinks (soda or others) that my child drinks. ^1^	Monitoring	0.925
**Weight Talk** (α = 0.89; MIIC = 0.72)	How often do you complain about your own weight where your child can hear you? ^2^	Weight Talk	0.875
	How often does your family comment on each other’s weight? ^2^	Weight Talk	0.900
	How often does your family talk about weight or dieting? ^2^	Weight Talk	0.899
**Distractions** (α = 0.83; MIIC = 0.62)	During a typical weekend day, how often is the television on during your child’s meals even if she or he is not watching it?		
	Breakfast ^2^	Distractions	0.857
	Snack ^2^	Distractions	0.870
	Dinner ^2^	Distractions	0.780
**Atmosphere of Meals** (α = 0.65; MIIC = 0.38)	How often would you say arguments about eating occur during dinner time? ^2^ (R)	Atmosphere of Meals	−0.798
	How often do other arguments, not about eating, occur during dinner time? ^2^ (R)	Atmosphere of Meals	−0.703
	How frequently is the evening meal an unpleasant or stressful time for your family. ^2^ (R)	Atmosphere of Meals	−0.739
**Modeling** (α = 0.74; MIIC = 0.49)	I try to eat healthy foods in front of my child, even if they are not my favorite. ^1^	Modeling	0.811
	My child learns to eat healthy snacks from me. ^1^	Modeling	0.621
	I eat food I want my child to eat. ^2^	Modeling	0.817
**Availability and Accessibility of Healthy Foods** (α = 0.65; MIIC = 0.48)	Do you have fruits and vegetables that your child likes available at home? ^2^	Availability/Accessibility of Healthy Foods	0.711
	How often is there fresh fruit on the counter, table, or somewhere else where your child can easily get to it? ^2^	Availability/Accessibility of Healthy Foods	0.800
**Planning, Preparation, and Attractive Presentation of Foods and Meals** (α = 0.69; MIIC = 0.38)	How often are there cut-up vegetables in the fridge for your child to eat? ^2^	Availability/Accessibility of Healthy Foods	0.569
	How often do you plan your family’s meals to provide a variety of food groups? ^2^	Planning/Preparation of Healthy Meals	0.723
	How often do you try to cook colorful (dark green, red, orange, purple) vegetables instead of potatoes or corn? ^2^	Planning/Preparation of Healthy Meals	0.705
	How often do you encourage vegetable consumption by preparing the vegetables in alternative ways? ^2^	Attractive Presentation of Healthy Foods	0.655
**Rules and Limits around Unhealthy Foods** (α = 0.64; MIIC = 0.26)	I place limits on the sweet or salty snacks (candy, ice cream, cake, potato chips) that my child eats. ^1^	Rules and Limits around Unhealthy Foods	0.528
	How often do you restrict (or try to restrict) your child’s access to sweetened beverages? ^2^	Rules and Limits around Unhealthy Foods	0.608
	If my child asks for sweetened beverages (including juice drinks or soda), I will give it to him or her. ^1^ (R)	Rules and Limits around Unhealthy Foods	−0.616
	How often do you allow your child to help himself or herself to snacks, including salty or sweet snacks, or candy when he or she is at home? ^2^ (R)	Rules and Limits around Unhealthy Foods	−0.742
	How often does your child eat in a bedroom? ^2^ (R)	Meal setting	−0.479
**Eating Area/Physical Space** (α = 0.61; MIIC = 0.44)	How often do you decorate your table with flowers? ^2^	Eating Area/Physical Space	0.829
	How often do you decorate your table with candles? ^2^	Eating Area/Physical Space	0.806

^1^ Response options offer a 5-point scale: strongly disagree, disagree, neutral, agree, and strongly agree. ^2^ Response options offer a 5-point scale: never, rarely, sometimes, often, and always. α = Cronbach’s α; MIIC = Mean inter-item correlation; and (R) = items that require reverse coding.

**Table 5:** Scores of food parenting practices for the full sample and according to children’s sex, age and weight status groups and correlations with body mass index (BMI) SD score (N = 184).

		Mean scores (SD) for Total Sample and by Weight Status and Differences between Weight Status Groups (One-Way ANOVA)							Pearson’s Correlation with BMIz
		Total Sample (N = 184)	Weigh Status ^1^						
			Normal (N = 92)	Overweight (N = 30)	Obesity (N = 22)	*F*	*p*	ƞ^2^	
**Coercive Control Practices**	Restriction	2.3 (1.0)	1.8 (0.8)	2.6 (1.0)	3.1 (1.1)	20.322	<0.001 ^a^	0.22	0.48 ***
	Soothing with food	1.6 (0.6)	1.6 (0.5)	1.6 (0.6)	1.8 (0.8)	2.110	0.124	0.03	−0.20
	“Clean plate” policy	3.5 (0.8)	3.5 (0.8)	3.5 (0.6)	3.2 (0.8)	2.129	0.123	0.03	−0.11
	Threats, bribes, pressure to eat	2.1 (0.7)	2.1 (0.7)	2.1 (0.6)	2.1 (0.7)	0.010	0.990	0.00	−0.14
**Autonomy Support Practices**	Encouragement and Reasoning	3.9 (0.6)	3.9 (0.6)	3.7 (0.7)	3.8 (0.9)	0.930	0.397	0.01	−0.14
	Praise	4.3 (0.5)	4.3 (0.5)	4.1 (0.4)	4.2 (0.6)	1.706	0.185	0.02	−0.03
	Nutrition Education	3.5 (1.0)	3.5 (1.0)	3.5 (0.9)	3.7 (0.9)	0.406	0.667	0.00	−0.08
	Guided choices: When and what food is eaten	2.6 (0.6)	2.7 (0.6)	2.5 (0.5)	2.5 (0.7)	1.724	0.182	0.02	−0.17 *
	Guided choices: Amount of food eaten	3.0 (0.7)	2.9 (0.7)	3.1 (0.5)	3.0 (0.8)	0.734	0.482	0.01	0.12
**Structure Practices**	Monitoring of unhealthy foods	4.1 (0.7)	4.1 (0.7)	4.1 (0.7)	3.9 (0.6)	0.904	0.407	0.01	−0.07
	Weight Talk	2.4 (1.0)	2.2 (0.9)	2.5 (0.9)	3.1 (1.2)	7.970	<0.001 ^b^	0.10	0.36 ***
	Distractions	3.1 (1.3)	2.9 (1.1)	3.3 (1.3)	3.9 (1.1)	5.941	<0.001 ^b^	0.08	0.31 ***
	Atmosphere of meals	4.0 (0.6)	4.0 (0.6)	4.1 (0.5)	3.9 (0.7)	0.532	0.589	0.01	−0.05
	Availability and accessibility of healthy foods	4.2 (0.6)	3.7 (0.7)	4.3 (0.6)	4.2 (0.9)	0.338	0.714	0.00	0.08
	Modeling	4.0 (0.7)	4.0 (0.8)	4.0 (0.7)	3.9 (0.6)	0.147	0.863	0.00	−0.01
	Planning, Preparation and attractive presentation of foods and meals	3.7 (0.7)	3.7 (0.7)	3.7 (0.6)	3.6 (0.7)	0.208	0.813	0.00	0.02
	Rules and Limits	4.0 (0.6)	4.0 (0.5)	4.1 (0.5)	3.8 (0.7)	1.949	0.146	0.03	−0.06
	Eating Area/Physical Space	1.7 (0.8)	1.7 (0.7)	1.9 (0.9)	1.6 (1.1)	0.972	0.381	0.01	0.02

BMIz = body mass index SD score. Note: We did not find the expected correlation with ‘Threats, bribes and pressure to eat’. ^1^ Missing data for 40 participants. ^a^ Tukey Honestly Significant Difference (HSD) test: overweight/obesity > normal weight; ^b^ Tukey HSD test: obesity > normal weight/overweight. * *p* < 0.05; *** *p* < 0.001.

**Table 6:** Correlations between each subscale of the HomeSTEAD family food practices survey and children’s Food Intake (N = 109).

		Fruit and Vegetables (Daily)	Sugar-Sweetened Beverages (Weekly)	Snacks (Weekly)	Sweets (Weekly)
**Coercive Control Practices**	Restriction	0.02	−0.11	−0.06	0.00
	Soothing with food	−0.14	0.11	0.19*	0.06
	“Clean plate” policy	0.01	−0.04	−0.04	−0.07
	Threats, bribes, and pressure to eat	−0.08	−0.01	−0.06	0.15
**Autonomy Support Practices**	Encouragement and Reasoning	−0.05	−0.08	0.03	0.08
	Praise	−0.11	−0.01	0.02	0.13
	Nutrition Education	0.02	−0.03	0.03	0.17
	Guided choices: When and what food is eaten	−0.15	0.21 *	0.14	0.08
	Guided choices: Amount of food eaten	0.10	0.01	−0.10	−0.11
**Structure Practices**	Monitoring of unhealthy foods	0.00	0.04	−0.05	−0.01
	Weight Talk	0.00	0.19	0.19 *	0.20 *
	Distractions	−0.10	0.11	0.05	0.25 ***
	Atmosphere of meals	−0.09	0.15	−0.05	−0.04
	Availability and accessibility of healthy foods	0.17	−0.06	−0.04	0.03
	Modeling	0.08	−0.40 ***	−0.20 *	−0.08
	Planning, Preparation and attractive presentation of foods and meals	0.08	0.03	−0.06	−0.05
	Rules and Limits	0.05	−0.45 ***	−0.20 *	−0.14
	Eating Area/Physical Space	−0.03	−0.00	0.00	−0.17

^1^ Missing data for 75 participants. Note: We did not find the expected significant correlations between children’s intake of fruit and vegetables with ‘guided choices’, ‘availability and accessibility of fruit and vegetables’, ‘nutritional education’, ‘encouragement’, ‘modeling’, ‘planning and preparation of healthy meals’, and ‘praise’. * *p* < 0.05; *** *p* < 0.001.

## Appendix A

**Table A1:** Excluded items during factorial analysis of the Portuguese version of the HomeSTEAD Survey for parents of 3–12 year-old children (N = 184).

Excluded Items	Original Factor	Reason for Exclusion
Autonomy Support Scales		
How often do you have to encourage your child to eat things s/he does not like (because those foods are good for him/her)? ^1^	Reasoning	Punctuated alone or not related
I negotiate with my child how much s/he can leave on his/her plate. ^2^	Reasoning	
This is a policy in our home: My child has to at least try or taste new foods. ^2^	Encouragement	
How often do you encourage vegetable consumption by making a game of eating vegetables or telling a story around vegetables? ^1^	Reasoning	Low Cronbach’s α
How often do you suggest your child have a fruit and vegetable at snack time? ^1^	Nutrition education	
I decide the times when my child eats his/her meals. ^2^	Guided choices: When food is eaten	Low Cronbach’s α
As a parent, I decide the kinds of food my child eats. ^2^	Guided choices: What food is eaten	
**Structure Scales**		
What do family dinners on weekdays look like at your home? ^3^	Family eating	Asymmetry/kurtosis
What do family dinners on weekends look like at your home? ^3^	Family eating	
When schedules allow, how often do you or another adult in your household sit and eat lunch at home with your child? ^1^	Family eating	Low factor loading
How often do you try not to eat unhealthy foods when your children are around? ^1^	Modeling	
How often do you drink soda (regular or diet) or other sweetened beverages at meals and snacks with your child? ^1^ (R)	Modeling	
How often do you bake, broil, barbeque, or steam food? ^1^	Planning/Preparation of Healthy Meals	
How often do you encourage vegetable consumption by serving vegetables in an interesting or attractive way? ^1^	Attractive presentation of foods	
Do you limit snacking to designated places in your home? ^1^	Meal setting	
How often do you decorate your table with a tablecloth? ^1^	Eating Area/Physical Space	
How often do you decorate your table with placemats? ^1^	Eating Area/Physical Space	
Dinner time is usually a pleasant time for the family. ^2^	Atmosphere of meals	Punctuated alone or not related
How often do you serve packaged, canned, or frozen dinners as the main dish of a meal? ^1^ (R)	Planning/Preparation of Healthy Meals	
When you serve potatoes, how often are they fried, like French fries or hash browns? ^1^ (R)	Planning/Preparation of Healthy Meals	
How often does your family eat fast food for the main meal each week? ^4^ (R)	Planning/Preparation of Healthy Meals	
How often do you restrict (or try to restrict) your child’s access to fruit juice? ^1^	Rules and Limits around Unhealthy Foods	
I insist my child eats meals at the table. ^1^	Meal setting	
How I eat does not particularly influence my child’s habits. ^2^ (R)	Modeling	Low Item-total
How often do you encourage vegetable consumption by serving vegetables hidden in other foods? ^1^	Attractive presentation of foods	

^1^ Response options offer a 5-point scale: never, rarely, sometimes, often, and always. ^2^ Response options offer a 5-point scale: strongly disagree, disagree, neutral, agree, and strongly agree. ^3^ Response options offer a 5-point scale: do not have dinners; child eats by him or herself or with other children; parent and children eat in different areas; one parent sits and watches child eat; and family members sit down and eat together. ^4^ Response options offer an 8-point scale ranging from seldom/never to 7 days per week.
